# Web of Science Journal Citation Report 2020: the Brazilian contribution to the “Medicine, General & Internal” category of the journal impact factor (JIF) ranking (SCI 2019)

**DOI:** 10.1590/1516-3180.2020.138419092020

**Published:** 2020-10-23

**Authors:** Álvaro Nagib Atallah, Maria Eduarda dos Santos Puga, José Luiz Gomes do Amaral

**Affiliations:** I MD, PhD. Full Professor and Head of the Discipline of Emergency Medicine and Evidence-Based Medicine, Universidade Federal de São Paulo (UNIFESP); and Director of Cochrane Brazil, São Paulo (SP), Brazil.; II MSc, PhD. Librarian, Evidence-Based Health Program, Universidade Federal de São Paulo (UNIFESP), São Paulo, Brazil; Director, Coordenadoria da Rede de Bibliotecas UNIFESP (CRBU), São Paulo (SP), Brazil; Information Specialist, Cochrane Brazil.; III MD, PhD. Full Professor, Discipline of Anesthesiology, Pain and Intensive Care, Department of Surgery, Universidade Federal de São Paulo (UNIFESP), São Paulo, Brazil.

To be included in the “Medicine, General & Internal” category of the Web of Science Journal Citation Report is a distinction for a select group of 165 journals that are considered to be the most influential in this category worldwide. This means standing shoulder-to-shoulder with giants like the New England Journal of Medicine, Lancet, JAMA, BMJ and Annals of Internal Medicine. Here, we wish to highlight the Brazilian presence among these journals.

The Web of Science (WoS) is a multidisciplinary database that provides access to 12,171 scientific journals, and it is edited under the responsibility of the company Clarivate Analytics. It is made available in Brazil through the journals portal of the Brazilian government's funding agency CAPES, to all institutions that are members of this portal.

The Web of Science Journal Citation Report (JCR) identifies and evaluates the most important science and social science journals worldwide and offers analysis on journal performance. It thus reflects the scientific and academic literature of the highest quality. The information that the JCR provides includes the total numbers of articles and citations, and data on the journals cited and those citing them, along with other analyses.^1^

*The journal impact factor* (JIF), published annually, has been eagerly awaited by researchers and journal editors around the world for the last 40 years. Its bibliometric mission has steered the scientific community towards editorial rigor and its results have influenced policies, partnerships, classifications and scientific analyses.

How is the JIF calculation made? The JIF of a given periodical for any given year (for example, 2020) is calculated from citations made during the previous year (2019, in this example) for items published in the two preceding years (2017 + 2018, in this example), divided by the number of citable items in these two years (2017 + 2018), published in that periodical.

The core collection of the Web of Science is composed of ten indexes containing thousands of periodicals. Three main citation indexes are responsible for the JIFs calculated from authors' citations, namely: Science Citation Index Expanded (SCI-Expanded), covering the years from 1900 to today (in which the São Paulo Medical Journal is included); Social Sciences Citation Index (SSCI), covering the years from 1900 to today; and Emerging Sources Citation Index (ESCI), covering the years from 2005 to today.^1^ A fourth major index, the Arts & Humanities Citation Index (A&HCI), covering the years from 1975 to today, is not used to generate an impact factor.

## “MEDICINE, GENERAL & INTERNAL” CATEGORY (SCI 2019)

The “Medicine, General & Internal” category of the SCI is formed by a total of 165 journals that are considered to be the group of periodicals within this field that are most influential worldwide. Table 1 shows the top ten of these 165 journals and also highlights the three Brazilian journals that also form part of this very select list, together with their respective JIFs.^.^^2^

**Table 1:** “Medicine, General & Internal” category rankings (SCI 2019)^2^

Ranking	“Medicine, General & Internal” journals	Journal impact factor
1.	New England Journal of Medicine	74.699
2.	Lancet	60.392
3.	JAMA – Journal of the American Medical Association	45.540
4	Nature Reviews Disease Primers	40.689
5.	BMJ – British Medical Journal	30.223
6.	Annals of Internal Medicine	21.317
7.	JAMA Internal Medicine	18.652
8.	PLOS Medicine	10.500
9.	Journal of Cachexia Sarcopenia and Muscle	9.802
10.	Cochrane Database of Systematic Reviews	7.890
**……**	**Three Brazilian journals are included among the 165 journals in this category:**	
**96.**	**Clinics**	**1.435**
**116.**	**São Paulo Medical Journal**	**1.044**
**128.**	**Revista da Associação Médica Brasileira**	**0.915**

The São Paulo Medical Journal is ranked 116^th^ in terms of its JIF, and the Cochrane Database of Systematic Reviews is ranked 10^th^, among the journals in which there is Brazilian participation. To plant its flag in the land of giants, the São Paulo Medical Journal has, since 1932, taken the firm stance of placing value on its authors and editors and has traced out clear aims in analyzing the international trends of major journals. Through this initiative, it has over recent years been attaining the desired recognition among its peers, in terms of JIF. The calculation is shown below.^2^

### Journal impact factor (JIF) calculation for the São Paulo Medical Journal (SPMJ)

**JIF calculation for SPMJ**

$$2019\text{ JIF}=\frac{166}{159}=1.044$$

**How is the JIF calculated?**

$$\text{ JIF}=\frac{\begin{array}{c} \text{Citations in 2019 of items published}\\ \text{in 2017}\left(108\right)+2018\left(58\right) \end{array}}{\begin{array}{c} \text{Number of citable items in}\\ \text{2017}\left(75\right)+2018\left(84\right) \end{array}}=\frac{166}{159}$$

In the 2020 edition of the JCR, among the 12,171 journals that it encompasses, 1,658 journals are classified as gold open access journals. Table 2 shows that the São Paulo Medical Journal is in this category, which means that all its articles are made public and accessible free-of-charge. This enables immediate use of them, thus contributing to the worldwide movement advocating open access.^2^

**Table 2:** Articles published in the São Paulo Medical Journal (SPMJ) in 2017 that were most cited in 2019; and the number of citations of each of these articles that were counted towards the journal impact factor. The arrow highlights the open padlock symbol that signifies that the SPMJ is a gold open access journal

Article	Number of citations
**Comparison of machine-learning algorithms to build a predictive model for detecting undiagnosed diabetes ELSA-Brasil: accuracy study** By: Olivera, Andre Rodrigues; Roesler, Valter; Iochpe, Cirano; Schmidt, Maria Ines; Vigo, Alvaro et al. Volume 135 Page: 234-246 Accession number: WOS:000406339500006 Document type: Article	9 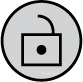 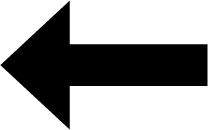
**Mortality due to noncommunicable diseases in Brazil, 1990 to 2015, according to estimates from the Global Burden of Disease study** By: Malta, Deborah Carvalho; Naghavi, Mohsen; Franca, Elisabeth; Xavier Abreu, Daisy Maria; Perillo, Rosangela Durso et al. Volume 135 Page: 213-221 Accession number: WOS:000406339500003 Document type: Article	8 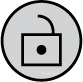
**The role of dietary fatty acid intake in inflammatory gene expression: a critical review** By: Rocha, Daniela Mayumi; Bressan, Josefina; Hermsdorff, Helen Hermana Volume 135 Page: 157-168 Accession number: WOS:000402009700011 Document type: Review	6 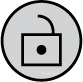
**Potential mechanisms linking probiotics to diabetes: a narrative review of the literature** By: Miraghajani, Maryam; Dehsoukhteh, Somayeh Shahraki; Rafie, Nahid; Hamedani, Sahar Golpour; Sabihi, Sima et al. Volume 135 Page: 169-178 Accession number: WOS:000402009700012 Document type: Review	5 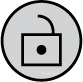
**Non-invasive brain stimulation and computational models in post-stroke aphasic patients: single session of transcranial magnetic stimulation and transcranial direct current stimulation. A randomized clinical trial** By: dos Santos, Michele Devido; Simis, Marcel; Bikson, Marom; Gagliardi, Rubens Jose; Cavenaghi, Vitor Breseghello et al. Volume 135 Page: 475-80 Accession number: WOS:000417223700010 Document type: Article	5 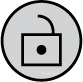
**Liver failure following biliopancreatic diversions: a narrative review** By: Cazzo, Everton; Pareja, Jose Carlos; Chaim, Elinton Adami Volume 135 Page: 66-70 Accession number: WOS:000398127000010 Document type: Review	5 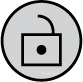
**Sensory-motor training versus resistance training among patients with knee osteoarthritis: randomized single-blind controlled trial** By: Gomiero, Aline Bassoli; Kayo, Andrea; Abraao, Marcelo; Peccin, Maria Stella; Grande, Antonio Jose et al. Volume 135 Page: 44-50 Accession number: WOS:000428567400007 Document type: Review	4 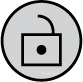

The Cochrane Database of Systematic Reviews is the periodical that is ranked tenth regarding its JIF. This ranking was achieved with participation from Brazil. Table 3 demonstrates that Brazil was in 16^th^ place as a contributor to its JIF, with 62 citations.

**Table 3:** Citations contributing to the journal impact factor (JIF) of the Cochrane Database of Systematic Reviews, according to country

Rank	Country	Citation count
1.	England	932
2.	Australia	416
3.	United States	358
4.	Canada	272
5.	Netherlands	157
6.	Scotland	142
7.	China Mainland	129
8.	Italy	126
9.	Germany (Fed Rep Ger)	122
10.	New Zealand	117
11.	Denmark	86
12.	Switzerland	86
13.	India	71
14.	Ireland	68
15.	Spain	65
16.	Brazil	62

The Federal University of São Paulo (Universidade Federal de São Paulo, UNIFESP) was the only Brazilian institution to contribute citations to the JIF of the Cochrane Database of Systematic Reviews. In the 2018 edition of the JCR, it was one of the top 50 institutions contributing to the JIF of this periodical, occupying 44^th^ place (Table 4).

**Table 4:** Citations contributing to the journal impact factor (JIF) of the Cochrane Database of Systematic Reviews, according to institutions and organizations. The arrow highlights the participation of the Federal University of São Paulo (Universidade Federal de São Paulo, UNIFESP)

Rank	Institution/organization	Citation count
41.	Queens University Belfast	29
42.	World Health Organization	29
43.	Hospital for Sick Children (SICKKIDS)	28
44.	Universidade Federal de São Paulo (UNIFESP) 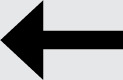	27
45.	University College London Hospital NHS Foundation Trust	27
46.	NHS Blood & Transplant	26
47.	University of Aberdeen	26
48.	South Australian Health & Medical Research Institute (SAHMRI)	25
49.	Vrije Universiteit Amsterdam	25
50.	Harvard University	24
51.	Norwegian Institute of Public Health (NIPH)	24
52.	Radboud University Nijmegen	24
53.	University of Milan	24

For journals to be accepted into this database, they are evaluated in terms of 28 criteria relating to quality, impact and editorial assessment. Thus, these journals have met these rigorous standards (Table 5).^3^

**Table 5:** Quality, impact and editorial evaluation criteria that journals need to meet in order to be included in the Web of Science

Quality criteria	Impact criteria
ISSN	Comparative analysis on citations
Title	Analysis on authors' citations
Journal publisher	Analysis on editorial board's citations
URL (online journals)	Significance of published content
Content access	
Presence of a peer review policy	
Contact details	
Academic content	**Editorial evaluation criteria**
Article titles and abstracts in English	Editorial board structure
Bibliographic information	Validity of declarations
Clarity of language	Peer review
Punctuality and publication volume per year	Content relevance
Website functionality/journal format	Funding details (acknowledgements)
Presence of ethics declarations in the journal	Adherence to community standards
Details of editorial board affiliations	Author distribution
Details of author affiliations	Auto-citations of the journal

There is no doubt that Brazilian science is well disseminated through several international databases and through the best journals. The São Paulo Medical Journal is proud to be among them.

It can also be asked, “Is being part of this scenario significant?” The response has to be affirmative. The São Paulo Medical Journal and the other two Brazilian periodicals (Clinics and Revista da Associação Médica Brasileira) that form part of this group are among the most influential journals in the world, with a JIF that can reach 74.699, which is very considerable and highly important.

The São Paulo Medical Journal provides an international window for health science results produced mostly by Brazilian researchers, with support from the recent and previous boards of directors of the São Paulo Medical Association (Associação Paulista de Medicina). We also send our compliments to the editors of the journals Clinics and Revista da Associação Médica Brasileira.

It is important to highlight the major contributions that have been made from Brazil to the Cochrane Library, which equal those of more-developed countries. Free-of-charge access to the Cochrane Library has been available for all Brazilians since 2001 (https://www.cochranelibrary.com/). This was achieved thanks to yearly efforts made by Cochrane Brazil's Director, with support from the Pan-American Health Organization (PAHO) via BIREME in the beginning and from CAPES and the CAPES portal more recently.
